# Purification and Characterization of Enterocins A, B, and a Novel High-Mass Bacteriocin from *Enterococcus lactis*-67 with Antilisterial Activity

**DOI:** 10.3390/antibiotics14090903

**Published:** 2025-09-06

**Authors:** Ezequiel Hernandez-Mendoza, Miguel Ángel Martínez-Téllez, Humberto González-Ríos, Emmanuel Aispuro-Hernández, María de la Cruz Paredes-Aguilar, Alexa Rubí-Soberanes, Etna Aida Peña-Ramos

**Affiliations:** 1 Centro de Investigación en Alimentación y Desarrollo, A.C. Carretera Gustavo Enrique Astiazaran Rosas 46, Col. La Victoria, Hermosillo 83304, Sonora, Mexico; ehernandez221@estudiantes.ciad.mx (E.H.-M.); norawa@ciad.mx (M.Á.M.-T.); hugory@ciad.mx (H.G.-R.); eaispuro@ciad.mx (E.A.-H.); alexarubi7@hotmail.com (A.R.-S.); 2 Centro de Investigación en Alimentación y Desarrollo, A.C., Área de Inocuidad Alimentaria, Varadero Nacional km. 6.6, as Playitas, Guaymas 85480, Sonora, Mexico; mparedes@ciad.mx

**Keywords:** bacteriocin, FPLC, biopreservative, *L. monocytogenes*

## Abstract

**Background/Objectives:** *Listeria monocytogenes* is a high-risk pathogen in the food industry involved in several outbreaks. Bacteriocins are natural-origin antimicrobial peptides or proteins that represent a good alternative to synthetic antimicrobials capable of inhibiting the growth of pathogens. This study aimed to purify and identify bacteriocins from the cell-free supernatant of *Enterococcus lactis*-67, which exhibits antagonistic activity against *L. monocytogenes*. **Methods:** Protein purification was performed by precipitation with ammonium sulfate, dialysis, and fast protein liquid chromatography. Active protein fractions were analyzed by SDS-PAGE and identified by mass spectrometry. **Results:** In addition to enterocins A and B, a novel 47 kDa bacteriocin with LysM and NlpC/P60 domains, on the N- and C-terminal regions, respectively, was identified. This enterocin has not been described for *Enterococcus* before. **Conclusions:** This study contributes to the identification of new natural and effective strategies for ensuring food safety.

## 1. Introduction

*L. monocytogenes* is one of the main threats in the food industry due to its ability to persist in adverse conditions, such as acidity, salinity, low temperatures, low oxygen, and its heat resistance, resulting in important economic losses due to food recalls and productivity diminution [1,2]. In addition, with the global trend towards “clean labels”, the food industry is seeking new alternatives to replace classic chemical/artificial additives that guarantee food safety [3]. Therefore, there is a growing interest in exploring the potential of new natural antimicrobials, such as bacteriocins.

Bacteriocins are antimicrobial peptides or proteins synthesized ribosomally by bacteria that possess the capability of exerting antagonistic activity against pathogenic bacteria, representing a promise for food safety [4]. The most studied are nisin and pediocin PA-1, produced by *Lactococcus lactis*, and *Pediococcus acidilactici*, respectively. The mechanisms of action of bacteriocins are varied and include pore formation in the cytoplasmic membrane, inhibition of cell wall biosynthesis, activation of enzymes involved in cellular lysis, and targeting of intracellular processes, such as DNA replication or protein synthesis. Additionally, certain bacteriocins can bind to receptor proteins associated with the cytoplasmic membrane, leading to depolarization of the membrane potential and cell death [5]. One of the most accepted and cited classifications of bacteriocins is that proposed by Alvarez-Siero et al., 2016 [6]. The authors established three classes taking into consideration their structure, molecular weight, post-translational modifications, thermostability, and other biological activities. In general, class I includes modified peptides also known as lantibiotics; class II includes unmodified active high peptides also known to have great antilisterial activity; class III includes thermolabile high molecular weight (>30 kDa) peptides [5]. They can be named based on their producer LAB, for example: pediocins or enterocins, those from *Pediococcus* or *Enterococcus*, respectively.

Recent studies have identified bacteriocin-like inhibitory substances (BLIS) from LAB isolated from fruits and vegetables such as apple (*Lactiplantibacillus plantarum*), mango (*Lactobacillus* spp.), lettuce (*L. lactis* LLH20), corn silage (*Pediococcus pentosaceus* LBM 18), and jalapeño peppers (*E. lactis*) with high antimicrobial potential [7,8,9,10]. However, these studies are limited since there is no follow-up by their BLIS purification and bacteriocin identification, which is essential to understand their properties and mechanism of action. Therefore, the screening and characterization of antimicrobial proteins, as well as their mechanism of action, remain crucial to elucidating potential applications.

In a previous study by Hernandez-Mendoza et al., 2024 [11], a BLIS produced by *Enterococcus lactis*-67, which was isolated from jalapeño peppers, showed a broad-spectrum antagonistic activity against pathogenic bacteria, including *L. monocytogenes*. This BLIS retained its antimicrobial activity at temperatures up to 121 °C for 15 min and in a wide pH range from 3 to 10, desirable characteristics in antimicrobials intended for the food industry. However, to develop specific applications, it is necessary to characterize and identify the bacteriocin(s) that exert the antimicrobial activity and to elucidate their mechanism of action. This study aimed to determine whether *E. lactis*-67 produces new bacteriocins with antilisterial activity.

## 2. Results

### 2.1. Purification and Identification of the Bacteriocins of E. lactis-67

**Figure 1:**
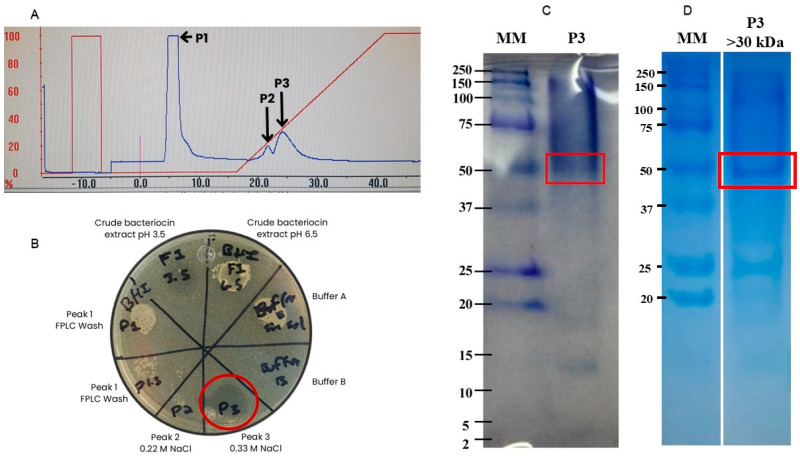
Purification of the BLIS-67 of *E. lactis*-67. (**A**): FPLC chromatogram from BLIS-67, dark arrow indicating the peak of interest at 0.33 M of NaCl. (**B**): Antagonistic activity of the eluted peaks (FPLC wash, 0.22 M and 0.33 M NaCl) against *L. monocytogenes*; red circle indicates the inhibition halo of the peak at 0.33 M NaCl. Buffers A and B, and crude bacteriocin extract were used as negative and positive controls, respectively. (**C**): SDS-PAGE, MM: Molecular mass marker, P3: Bioactive bacteriocin fraction (0.33 M), in red square is the high molecular mass protein band cut from the gel for MS/MS analysis. (**D**): SDS-PAGE, MM: Molecular mass marker, P3 > 30 kDa: Bioactive bacteriocin fraction (0.33 M) containing proteins with molecular mass >30 kDa, in red square is the high molecular mass protein.

The BLIS-67 was purified by FPLC, and only two peaks were detected, one at 0.22 and another at 0.33 M of NaCl (Figure 1A). Figure 1B shows the evaluation of the antagonistic activity against *L. monocytogenes* of peaks at 0.22 M (P2) and 0.33 M (P3) of NaCl, as well as the wash peak (P1). Moreover, Buffer A and B, as well as crude bacteriocin extract (BLIS-67), were used as negative and positive controls, respectively. In this Figure, it can be observed (red circle) that only P3 showed antagonistic activity. Figure 1C shows two main protein bands in the active fraction obtained by ion exchange chromatography: one with an approximate experimental molecular mass of 50 kDa and another of 14 kDa, according to the protein molecular weight marker. Figure 1D shows the protein band of interest after being separated using a 30 kDa cut-off microfilter. It is worth mentioning that both filtered fractions, <30 and >30 kDa, retained their antagonistic activity against *L. monocytogenes*.

### 2.2. Identification of the Amino Acid Sequences of the Bacteriocins of E. lactis-67

Mass spectrometry analysis provided the amino acid sequences of the identified bacteriocins (Table 1). The ≈50 kDa in-gel band analysis identified a bacteriocin composed of 473 amino acids with a theoretical molecular mass of 46.9 kDa, which was consistent with the electrophoretic results. The identified bacteriocin was designated as enterocin HM (EHM). In addition, analysis revealed nine unique peptide sequences, which are representative of the domains present in the aminoacidic sequence. Furthermore, the amino acid sequence of EHM was searched against the NCBI and UniProt databases, and it was found to be homologous to the LysM domain protein from *Pediococcus acidilactici* DSM 20284. On the other hand, mass spectrometry analysis of the enriched < 30 kDa fraction detected three unique peptides that matched (100%) with a bacteriocin predicted for *E. faecium* (accession number A0A9X3XT33, UniProt), with a reported molecular weight of 7.46 kDa, that shares high identity to enterocin B. Additionally, one unique peptide was detected and matched (100%) to a class II bacteriocin of 6.94 kDa inferred for *E. faecium* (accession number A0A9X1GA65, UniProt). This protein also contained the highly conserved YGNGV motif and shares high identity with enterocin A. It has been reported that enterocins A and B can act synergistically and improve their antibacterial activity. Additionally, they can form a heterodimer stabilized by Van der Waals and electrostatic interactions [12]. Interestingly, the sum of the molecular weights of the enterocins identified in our work corresponds to ≈approximately 14 kDa, which is the size of the protein band that was not identified using the in-gel digestion strategy.

### 2.3. Bioinformatic Analyses of the Amino-Acid Sequence of EHM from E. lactis-67

**Figure 2:**
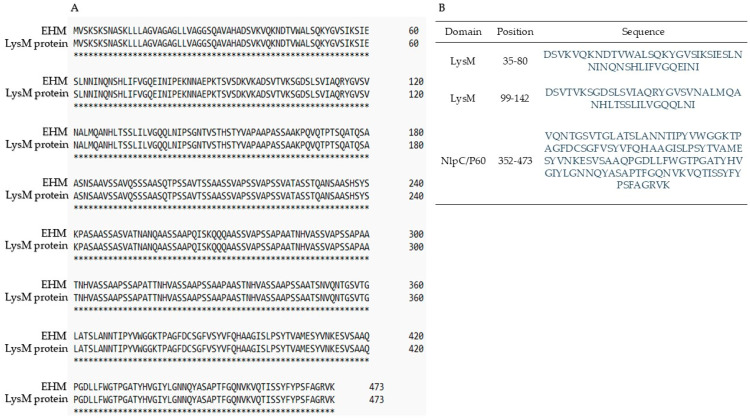
(**A**): Sequence alignment between EHM from *E. lactis*-67 and LysM protein from *P. acidilactici* obtained from Clustal Omega. Asterisks (*) indicate amino acid match. (**B**): LysM and NlpC/P60 domains’ positions within both sequences.

BLASTp analysis of the amino-acid sequence of EHM (47 kDa) yielded a high percentage of identity with several sequences reported as peptidase proteins of the C40 family of *P. acidilactici* species, with a 100% query cover. The C40 family peptidase protein with 100% identity (GenBank: WP_022831970.1) was searched in the UniProt database, where it was identified as “an inferred from homology protein” with a lysozyme motif domain (LysM) previously reported in *P. acidilactici* (UniProt: E0NEA3). This sequence was aligned to the amino acid sequence of EHM from *E. lactis*-67 using Clustal Omega software (version 1.2.4), and it was observed that both sequences were completely identical (Figure 2A). Hence, EHM had a 100% identity with the LysM protein reported for *P. acidilactici*. Moreover, the LysM protein of *P. acidilactici* presents three domains in its sequence: two LysM domains at the N-terminal, and one NlpC/P60 domain at the C-terminal. These three domains matched in position with the unique peptide sequences detected in the EHM protein from *E. lactis*-67. Therefore, EHM from *E. lactis*-67 also have two LysM domains and an NlpC/P60 domain (Figure 2B).

Subsequently, the *Pediococcus* genus was excluded from a subsequent BLASTp analysis, and the search was limited only to the *Enterococcus* genus to evaluate whether other *Enterococci* species produced similar proteins. It was observed that EHM shares more than 50% of identity with other *Enterococcus* species proteins of the Nlpc/P60 family. Then, the proteins with high percentages of identity, which were from *E. mediterraneensis* (GenBank: WP_122646106.1), *E. faecium* (GenBank: WP_167778979.1), and *E. lactis* (GenBank: WP_277904299.1), were selected for their alignment in Clustal Omega with the EHM sequence. The highest similarity was observed at the C-terminal, where the NlpC/P60 domain was in EHM. In contrast, the N-terminal, which contains the LysM domains, showed lower similarity between the compared sequences.

## 3. Discussion

The demand for new natural antimicrobial compounds with the potential to be used in the food industry against relevant pathogenic bacteria, such as *L. monocytogenes*, has created the need for the identification of new bacteriocins [13]. Previous studies have documented the antagonistic activity of enterocins against *L. monocytogenes*. Among these are enterocins A and B, synthesized by *E. faecium*, which can inhibit the growth of *L. monocytogenes* in meat products such as ham, chicken breast, and sausages [14]. As for *E. lactis*, Ben Braïek et al., 2018 [15] reported that this species can synthesize enterocins A, B, and P. Additionally, Shastry et al., 2021 [16] identified a new enterocin (R5) from *E. lactis* RS5, which presented antagonistic activity against *L. monocytogenes*. In the present study, the antagonistic activity of *E. lactis*-67 against *L. monocytogenes* may be attributed to the production of enterocins A, B, and a new high-molecular-mass enterocin (EHM). Therefore, its characterization provides novel information and expands the knowledge about the enterocins that this genus can synthesize.

Mass spectrometry analysis identified the amino-acid sequences of the enterocins responsible for the antimicrobial activity of *E. lactis*-67 against *L. monocytogenes*. Particularly, EHM had a high molecular mass (47 kDa) in comparison to other enterocins with molecular masses ≤ 10 kDa, previously reported [17]. Therefore, EHM may be classified as a class III bacteriocin according to Alvarez-Siero et al., 2016 [6]. Previous studies have reported other high molecular mass enterocins with antimicrobial activity against *L. monocytogenes,* such as enterolysin A (34.5 kDa) produced by *E. faecalis* LMG 2333, bacteriocin EF478 (45 kDa) from *E. faecalis* 478, enterocin 12a from *E. faecium* (65 kDa), and enterocin-7 (65 kDa) from *E. faecium* ICIS 7 [18,19,20,21].

After analysis of the 473 amino-acid sequence of EHM, BLASTp found 100% identity with the LysM domain protein of *P. acidilactici* (GenBank accession number: EFL96562.1; UniProt: E0NEA3). This *P. acidilactici* protein has three domains in its sequence: two LysM domains at the N-terminal and an NlpC/P60 domain at the C-terminal. Moreover, the unique peptide sequences identified in *E. lactis*-67 align in position with the domains of the *P. acidilactici* protein, suggesting that EHM possesses the exact structural domains that can be related to its antagonistic activity against *L. monocytogenes*. Proteins with NlpC/P60 domains act as hydrolases of peptidoglycan, the major component of the bacterial cell wall [22]. This domain is widely distributed in prokaryotes and is often found in larger proteins containing other peptidoglycan-binding associated domains, such as the LysM domain [23]. The LysM domain is a β-1,4 glycosidic binding domain between N-acetylglucosamine and N-acetylmuramic acid, which are the main components of peptidoglycan. The LysM domain is widely distributed among prokaryotes and is frequently found in bacterial lysins, bacterial peptidoglycan hydrolases, and peptidases [24].

The presence of the LysM and NlpC/P60 domains in the sequence of EHM indicates that its possible mechanism of action against *L. monocytogenes* involves the binding of EHM to the peptidoglycan of the target cell wall, mediated by the LysM domain, followed by its degradation through the hydrolytic activity of the NlpC/P60 domain, resulting in bacterial lysis. This mechanism of action is characteristic of bacteriocins from class IIIa [25]. Nilsen et al., 2003 [18] reported that Class III enterolysin A from *E. faecalis* LMG 2333 acts by degrading the cell wall and causing bacterial lysis. However, it contains different domains corresponding to endopeptidases of the M37 protease family, which act on the peptide bonds in the peptidoglycan side chains.

On the other hand, BLASTp analysis of EHM, excluding sequences from the *Pediococcus* genus, to evaluate the presence of similar proteins in other *Enterococcus* species, showed that EHM had more than 50% similarity with other proteins in other *Enterococcus* species. However, this similarity is mainly due to the presence of the NlpC/P60 domain, as no LysM domains were detected in the analyzed sequences.

Moreover, LysM domains have been reported in *Enterococcus*, like in the peptidoglycan hydrolase AtlA from *E. faecalis* [26] and proteins from *E. faecium*, presenting the characteristic highly conserved motif: DEVYTVKSGDSL [27,28]. Likewise, the LysM domain of EHM presented a similar sequence, DSVYTVKSGDSL (unique peptide 6, Table 1), which partially matched the highly conserved motif of *E. faecium*. However, the *Enterococcus* protein sequences to which EHM was compared did not present the LysM domains in their sequences. Therefore, it can be suggested that EHM from *E. lactis*-67 is novel and has not been previously reported within the *Enterococcus* genus.

The reason why EHM from *E. lactis*-67 presented a 100% match with the protein from *P. acidilactici* could be explained by the transfer of bacteriocin genes between different genera. Todorov et al., 2010 [29] reported the presence of pediocin genes in a bacteriocin (ST5Ha) from *E. faecium*, which is unusual since most pediocins are produced by *Pediococcus* species. Hammi et al., 2019 [30] reported the native production of pediocin PA-1 by *E. faecium* E16. According to these authors, genes involved in pediocin production are highly conserved but can be found in bacteria outside the genus *Pediococcus*, suggesting a possible genetic exchange between species. The natural production of bacteriocins across genera and even species is a relatively rare phenomenon. To the best of our knowledge, this is the first report on the presence of the LysM protein from *P. acidilactici* in another genus and species outside *Pediococcus*.

The production of multiple bacteriocins by *E. lactis*-67 represents an adaptive advantage for this strain. The different target specificity of these enterocins (A, B, and EHM), suggests that they may possess different mechanisms of action, and therefore their combined use as biopreservatives may contribute to reducing the frequency at which resistant bacterial populations develop. This study provides new insights into the natural occurrence of LysM from *P. acidilactici* among lactic acid bacteria. It offers evidence that *E. lactis*-67 produces enterocins A, B, and a novel high-mass enterocin (EHM). Elucidation of their mechanisms of action and optimization of bacteriocin production will be crucial for evaluating their potential use in the food industry as natural antimicrobial compounds to control foodborne pathogens.

## 4. Materials and Methods

### 4.1. Bacteria Strains Conditions, Activation, and Antagonistic Activity

The bacteria used in the study were cryopreserved at −80 °C in brain-heart infusion broth (BHI) (DIFCO, Detroit, MI, USA) with 15% glycerol. *E. lactis*-67, previously identified by Hernandez-Mendoza et al., 2024 [11], was reactivated by inoculating 0.1 mL into Man, Rogosa, and Sharpe (MRS) broth (DIFCO, Detroit, MI, USA) at pH 7.0 ± 0.2 at 37 °C for 24 h. Two transfers were performed under the same conditions and kept refrigerated at 4 °C until use. *L. monocytogenes* ATCC 7644 was reactivated by inoculating 0.1 mL in BHI broth and incubating at 37 °C for 24 h. The strains were kept refrigerated at 4 °C throughout the study, with monthly transfers. On the day of the assay, fresh overnight-grown cultures were centrifuged (8000× *g*, 10 min at 4 °C), and the resulting pellets from each strain were washed twice with sodium phosphate buffer (20 mM, pH 6.5 ± 0.2). These pellets were then used in the experiments.

The antagonistic activity of *E. lactis*-67 against *L. monocytogenes* was evaluated by the spot-on-lawn method described by Hernandez-Mendoza et al., 2024 [11]. Briefly, 20 μL of *E. lactis*-67 were deposited on MRS agar plates and allowed to dry. Subsequently, the *L. monocytogenes* inoculum (optical density at 600 nm of 0.1) was mixed with BHI soft agar and poured onto MRS plates containing *E. lactis*-67. After solidification of the agar, the plates were incubated at 37 °C for 24 h. Antagonistic activity against the pathogenic bacterium was confirmed by the formation of clear inhibition halos around the *E. lactis*-67 colonies.

### 4.2. Semi-Purification of the BLIS of E. lactis-67

*E. lactis*-67 was grown in 1000 mL of MRS broth at 37 °C for 18 h. The overnight fresh culture was centrifuged (8000× *g*, 20 min at 4 °C), and the cell-free supernatant (CFS) was collected. Subsequently, to obtain BLIS-67, protein precipitation was performed according to Rasheed et al., 2020 [31] with several modifications. Ammonium sulfate was gradually added into the CFS up to 50% (*w*/*v*) saturation with continuous stirring at 4 °C until a homogenized solution was obtained. Then, the saturated solution was centrifuged (4000× *g*, 20 min at 4 °C), and the resulting pellet (BLIS-67) was resuspended in 15 mL of sodium phosphate buffer (20 mM, pH 6.0 ± 0.2). The BLIS-67 was desalted by dialysis using a 3.5 kDa molecular mass cutoff membrane (Spectra/Por, Spectrum, Stamford, CT, USA) with the same sodium phosphate buffer. The desalted BLIS-67 was microfiltered and sterilized with 0.22 µm membranes (Milipore, Cork, Ireland) and stored at 4 °C for further analysis. The antagonistic activity against *L. monocytogenes* was tested by the spot-on-lawn assay.

### 4.3. Purification and Molecular Mass Estimation of the Bacteriocin from E. lactis-67

Purification of the resulting semi-purified BLIS-67 was performed by fast protein liquid chromatography (FPLC) using a cation-exchange HiTrap SP Sepharose Fast Flow column (Cytiva, Marlborough, MA, USA) following the method described by Rasheed et al., 2020 [31] with modifications. The column was activated with 5 volumes of buffer A (sodium phosphates at 20 mM at pH 3.5) followed by a second wash with 5 volumes of buffer B (sodium phosphates at 20 mM at pH 3.5 saturated at 1 M NaCl), and finally a third wash with 5 volumes of buffer A. Once the column was activated, 2 mL of the BLIS-67 (pH 3.5) was injected into the FPLC, and elution was performed with a linear gradient of 0–100% buffer B at a flow rate of 3 mL/min. The eluted protein fractions were detected at 280 nm, collected, and evaluated for antimicrobial activity against *L. monocytogenes* by the spot-on-lawn assay. The protein fraction with antimicrobial activity was stored at 4 °C for further analysis.

For SDS-PAGE analysis, the protein fraction with antimicrobial activity was concentrated by precipitation with pure acetone [32]. Subsequently, the sample was resuspended in 50 μL of ultrapure water and prepared by mixing 24 μL of the concentrated protein fraction with 6 μL of 5× SDS-DTT loading buffer and heated at 95 °C for 5 min. After incubation time, the sample was immediately transferred to ice and loaded into a Tris-Tricine-SDS 14% polyacrylamide gel and run in a Mini-Protean Tetra electrophoresis system (Bio-Rad, Hercules, CA, USA) at 15 mV for 2 h. The Precision Plus Protein standard from Bio-Rad was loaded and used as a molecular mass marker. After electrophoresis, the gel was stained with 1% Coomassie Brilliant Blue R-250 to visualize the protein bands.

### 4.4. Identification of the Bacteriocin from E. lactis-67 by Liquid Chromatography Tandem Mass Spectrometry (LC–MS/MS)

Bacteriocin identification was performed according to Rivas-Mercado et al., 2025 [33]. The SDS-PAGE-separated protein bands were excised from the gel and prepared for mass spectrometry analysis. Sample preparation was done by adding 10 mM dithiothreitol (DTT) with 55 mM iodoacetamide (IAA; Sigma-Aldrich, Saint Louis, MO, USA) for 30 min at 60 °C. Then, the sample’s peptides were obtained through an overnight in-solution digestion with trypsin (Promega, Madison, WI, USA). The resulting peptides were desalted with C18-Ziptips (Millipore, Burlington, MA, USA) and concentrated in a SpeedVac (Savant SPD1010, Thermo Fisher Scientific, Waltham, MA, USA). The desalted and concentrated peptides were resuspended in 20 μL of Milli-Q water for quantification and analysis. Based on results obtained with the in-gel digestion strategy, a second approach was used to increase the concentration of lower molecular weight proteins in the active fraction and improve the signal-to-noise ratio of the LC–MS/MS spectra. Briefly, low molecular weight proteins in the 0.33 M NaCl eluted peak were enriched using 30 kDa cut-off Amicon Ultra-4 microfilters (Millipore, USA). The filtrate fraction containing <30 kDa proteins was desalted and concentrated with 3 kDa cut-off Amicons (Millipore, USA) using buffer A. Afterwards, the sample was freeze-dried and prepared for mass spectrometry analysis as described previously. Moreover, fractions < 30 and >30 kDa were tested for their antagonistic activity against *L. monocytogenes* by the spot-on-lawn assay.

An LC–MS system (Ultimate 3000 Dionex–LTQ Orbitrap Velos, Thermo Fisher Scientific, USA) and a C18 capillary column (Acclaim PEPMAP, Thermo Fisher Scientific, USA) were used to analyze each sample. The system was calibrated by using a mixed ion positive ion calibration solution (LTQ ESI, Pierce, Thermo Fisher Scientific, USA). Then, 0.5 μg of each sample was injected into the system independently. The peptides were separated using a gradient of 4 to 85% of acetonitrile with 0.1% formic acid for 120 min, maintaining a flow rate of 300 nL/min. All spectra were acquired in positive-ion mode. The acquisition method involved a dynamic exclusion set to a maximum of 500 ions and 70 s for the exclusion duration. Full-scan MS spectra from *m*/*z* 400 to 1600 were acquired at a resolving power of 60,000, a 3.0 Da isolation width, and 35 arbitrary normalized collision energy units. Collision-induced dissociation and high-energy collision-activated dissociation were alternately used for fragmentation.

### 4.5. Data and Bioinformatic Analysis

Scaffold (Proteome Software, Inc., Portland, OR, USA, version 5.3.3) was used to probabilistically validate protein identifications derived from MS/MS sequencing results using the X!Tandem [34] and ProteinProphet computer algorithms [35].

Once the amino-acid sequences of the *E. lactis*-67 bacteriocins were identified, a peptide search was performed in the UniProt database. After that, bacteriocins were analyzed by protein-to-protein BLAST (BLASTp) software (https://blast.ncbi.nlm.nih.gov/Blast.cgi?PROGRAM=blastp&PAGE_TYPE=BlastSearch&BLAST_SPEC=&LINK_LOC=blasttab&LAST_PAGE=blastp; accessed on 1 August 2025) (NCBI/NHI) to identify regions of similarity to other biological sequences. For each BLAST analysis, an unrestricted search was performed first, followed by a second search restricted exclusively to the genus *Enterococcus*. Amino-acid sequence with the highest percentage of identity was selected and aligned using Clustal Omega software (EMBL-EBI) for comparison.

## 5. Conclusions

*E. lactis*-67 produces a new 47 kDa enterocin, designated as EHM, which belongs to the peptidase C40 family with two LysM and one NlpC/P60 domain. Moreover, *E. lactis*-67 is also capable of producing enterocins A and B. The results of this study demonstrate the potential of *E. lactis*-67 enterocins as a new, natural alternative for controlling *L. monocytogenes* and ensuring food safety, while also contributing to the understanding of bacteriocin diversity. Future studies should focus on the purification of EHM, and enterocins A and B within the active fraction (P3), as well as the evaluation of the antagonistic activity of each individual enterocin to clarify whether they act synergistically. Moreover, it is recommended to continue with in situ simulation studies to evaluate the antimicrobial activity of *E. lactis*-67’s enterocins once applied in a food matrix.

## Figures and Tables

**Table 1:** Identified bacteriocins from the purified BLIS-67 of *E. lactis*-67 by tandem mass spectrometry.

Identified Protein (Accession Number *)/ Theoretical Molecular Mass (kDa)	Amino Acid Sequence	Unique Peptide Sequences
EHM (E0NEA3)/ 46.9	MVSKSKSNASKLLLAGVAGAGLLVAGGSQAVAHADSVKVQKNDTVWALSQKYGVSIKSIESLNNINQNSHLIFVGQEINIPEKNNAEPKTSVSDKVKADSVTVKSGDSLSVIAQRYGVSVNALMQANHLTSSLILVGQQLNIPSGNTVSTHSTYVAPAAPASSAAKPQVQTPTSQATQSAASNSAAVSSAVQSSSAASQTPSSAVTSSAASSVAPSSVAPSSVATASSTQANSAASHSYSKPASAASSASVATNANQAASSAAPQISKQQQAASSVAPSSAPAATNHVASSVAPSSAPAATNHVASSAAPSSAPATTNHVASSAAPSSAAPAASTNHVASSAAPSSAATSNVQNTGSVTGLATSLANNTIPYVWGGKTPAGFDCSGFVSYVFQHAAGISLPSYTVAMESYVNKESVSAAQPGDLLFWGTPGATYHVGIYLGNNQYASAPTFGQNVKVQTISSYFYPSFAGRVK	1.(K)VQKNDTVWALSQK(Y) 2.(K)NDTVWALSQK(Y) 3.(K)SIESLNNINQNSHLIFVGQEINIPEK(N) 4.(K)SIESLNNINQNSHLIFVGQEINIPEKNNAEPK(T) 5.(K)TSVSDKVK(A) 6.(K)SGDSLSVIAQR(Y) 7.(K)ESVSAAQPGDLLFWGTPGATYHVGIYLGNNQYASAPTFGQNVK (V) 8.(K)VQTISSYFYPSFAGR(V) 9.(K)VQTISSYFYPSFAGRVK(-)
Enterocin B (A0A9X3XT33)/ 7.46	MQNVKEVSVKEMKQIIGGENDHRMPNELNRPNNLSKGGAKCGAAIAGGLFGIPKGPLAWAAGLANVYSKCN	1.(R)MPNELNRPNNLSK(G) 2.(K)CGAAIAGGLFGIPK(G) 3.(K)GPLAWAGLANVYSK(C)
Enterocin A (A0A9X1GA65)/ 6.94	MKHLKILSIKETQLIYGGTTHSGKYYGNGVYYTKNKCTVDWAKATTCIAGMSIGGFLGGAIPGKC	1.(K)ATTCIAGMSIGGFLGGAIPGK Pediocin-like conserved motif: YGNGV

* Accession number according to UniProt.

## Data Availability

All data generated or analyzed during this study are available upon request.
